# DOAC in the treatment of cancer-associated venous thromboembolism: a retrospective cohort study beyond the guidelines

**DOI:** 10.1007/s00432-022-04535-8

**Published:** 2022-12-28

**Authors:** Mattia Cominacini, Silvia Suardi, Giulia Ferrari, Roberto Ciresa, Federica Tosi, Sergio De Marchi, Maria Teresa Valenti, Luca Dalle Carbonare

**Affiliations:** 1 Department of Medicine, Section of Internal Medicine, University of Verona, Piazzale Scuro, 10, Policlinico G.B. Rossi, 37134 Verona, Italy; 2 Department of Neurosciences, Biomedicine and Movement Sciences, University of Verona, 37134 Verona, Italy

**Keywords:** Anticoagulants, Cancer, DOAC, Safety, Thromboembolism

## Abstract

**Background:**

The emerging use of direct oral anticoagulants (DOAC) in the management of cancer-associated venous thromboembolism (CAT) is significantly improving therapeutic adherence and quality of life. Despite this, many conditions can restrict the therapeutic index of these drugs. For all these reasons the latest guidelines recommend the use of heparins in the treatment of CAT as the preferred treatment in some clinical settings.

**Objectives:**

We evaluated the efficacy and the safety of DOAC, in terms of recurrent venous thromboembolism (VTE) and major bleeding (MB), as a composite primary outcome. Mortality and clinically relevant non-major bleeding (CRNMB) were evaluated as secondary outcomes.

**Methods:**

We performed a retrospective study on 209 patients to compare the effects of DOAC versus heparins for the treatment of CAT. 127 patients with a high bleeding risk neoplasia were enrolled.

**Results:**

A primary-outcome event occurred in 11.3% of patients treated with heparins and in 10.5% treated with DOAC (Relative Risk 0.92; 95% CI 0.42–2.01, *p* = 0.84). Recurrent VTE occurred in 6.1% in the heparins group and in 8.4% in the DOAC group (RR 1.37; 95% CI 0.51–3.64, *p* = 0.52). MB occurred in 5.2% in the heparins group and in 2.1% in the DOAC group (RR 0.40; 95% CI 0.08–1.93, *p* = 0.25).

**Conclusions:**

DOAC seem to be as effective and safe as heparins in the treatment of CAT. Most bleeding events occurred in patients with high-risk bleeding neoplasms regardless of the type of anticoagulant. Considering the characteristics and satisfaction of patients using DOAC in this setting, this approach should be considered as a first choice.

## Introduction

Cancer-associated thrombosis (CAT) is a common complication in the history of cancer disease (Blom et al. 2005). It represents the second leading cause of death, after cancer itself. Furthermore, CAT therapy is associated with higher rates of recurrent thrombosis and/or major bleeding compared with the non-cancer venous thromboembolism (VTE) (Mulder et al. 2021; Khorana et al. 2007b, 2013; Khorana et al. 2007a). Treatment of CAT is often complex and comprises initial treatment, long-term treatment, treatment within 6 months, treatment beyond 6 months, treatment of recurrent VTE, and treatment in special situations (Farge et al. 2019; Key et al. 2021; Streiff et al. 2021). The choice of antithrombotic therapy, the selection of anticoagulants, the duration of anticoagulation phase, the choice of adjuvant therapy, and adjustment of regimen in special situations are the major problems in the treatment of CAT. Finally, the multiplicity of types of cancer, the disease stage and the imbricated cancer treatment can represent additional challenges for the clinician (Farge et al. 2019; Key et al. 2021; Streiff et al. 2021). Historically, heparin has been the preferred choice for the treatment of CAT. Following the publication of the 2018 guidelines, edoxaban and rivaroxaban replaced heparin as preferred treatment. In the 2020 guidelines, apixaban was added, based on the positive results obtained in large randomized clinical trials (Raskob et al. 2017; Young et al. 2018; Agnelli et al. 2018).

In the Hokusai-VTE study, Edoxaban was non-inferior to dalteparin in terms of recurrence of VTE, with a similar risk of bleeding, except for gastrointestinal bleeding (increased risk) (Raskob et al. 2017); in the SELECT-D study, rivaroxaban was superior to dalteparin in terms of VTE recurrence, but higher CRNMB compared with dalteparin (Young et al. 2018). In the CARAVAGGIO study, apixaban resulted non-inferior to dalteparin in terms of relapses, without an increased risk of bleeding (Agnelli et al. 2018).

Despite the indisputable advantages of DOAC in terms of quality of life and therapeutic adherence (Wojtukiewicz et al. 2020), in the “real life”, uncertainties remain on the use of DOAC, especially for the bleeding risk in patients with gastrointestinal cancers (Mai et al. 2020 Oct; Elbadawi et al. 2021) and the potential drug-to-drug interactions with specific anticancer therapies (Harskamp et al. 2019) such as strong P-glycoprotein or tyrosine kinase or CYP3A4 inhibitors/inducers. The latest ASCO, ITAC and NCCN guidelines agree on many aspects of CAT treatment, but differ on few relevant issues (Raskob et al. 2017; Young et al. 2018; Agnelli et al. 2018). Currently, anticoagulant therapy for cancer patients should be individualized with multidisciplinary follow-up and frequent reassessment.

## Materials and methods

We conducted a retrospective cohort study, patients were enrolled between January 2018 and January 2022. The study included adult patients with active cancer and/or undergoing chemotherapy, with a recent diagnosis of VTE: pulmonary embolism (PE), deep venous thrombosis (DVT), peripherally inserted central catheter (PICC)-DVT and atypical-deep venous thrombosis (VTE-A), therefore candidates for anticoagulant treatment. We classified active cancer patients as follows: patients with cancer diagnosed within the previous 6 months, patients with regionally advanced or metastatic cancer, patients with cancer for which treatment was initiated within 6 months prior to randomization and patients with haematological malignancies not in complete remission.

All CAT events were diagnosed by computed tomography pulmonary angiography (CTPA) or by ultrasonography. Patients with gastric cancer were excluded.

During the outpatient visit, a complete medical history was collected, physical examination was performed, daily living abilities were assessed using the ECOG performance status scale and blood chemistry tests results were viewed.

The choice of the type and dose of the drug was made by the physician. An outpatient clinical and biochemical evaluation was repeated at 1, 3, 6 and 12 months, respectively in each type of therapy. A total of 209 patients were enrolled. Characteristics of patients are reported in Table 1. 95 patients were treated with DOAC, respectively 68 with edoxaban (60 mg/day or 30 mg/day in patients who met the criteria for dose reduction, after at least 5 days of parenteral anticoagulation) and 27 with rivaroxaban (at the initial dose of 15 mg BID for 3 weeks or parenteral anticoagulation, followed by 20 mg/day). 114 patients were treated with heparins of which 45 with enoxaparin and 69 with fondaparinux, both with an anticoagulant dose based on their body weight and their glomerular filtrate (GFR) (Table 2). Out of the 68 patients receiving edoxaban, 10 met the criteria for dose reduction to 30 mg. In the fondaparinux group, 10 patients also met the criteria for dose reduction to 5 mg. Due to the publication date of the Caravaggio study being subsequent to the start of our study, apixaban treatment hasn’t been considered in our study. Edoxaban and rivaroxaban blood levels were measured with specific kits calibrated for anti F-Xa activity (HemosIL® Liquid Anti-Xa, Werfen). Blood samples were taken before to the administration of the daily dose of the drug. Statistical analysis was assessed by two-tailed Student’s paired-test, relative risk (for events with prevalence < 20%) and odds ratio (for events with prevalence > 20%). Differences were considered positive when *p* < 0.05. We used SPSS for Windows, version 22.0 (SPSS Inc., Chicago, IL, USA) to analyze the data.

**Table 1:** Clinical characteristics of the direct oral anticoagulants (DOAC) group and heparins one

	DOAC (*n* = 95)	HEPARINs (*n* = 114)
Male (*n*)	50	62
Female (*n*)	45	52
Mean age (years)	68 ± 10	69 ± 11
Body weight (Kg)	76.3 ± 31.5	79.1 ± 32.2
Follow-up (months)	6 ± 6	6 ± 6
Eastern cooperative oncology group (ECOG) performance status 0–1	79	79
Eastern cooperative oncology group (ECOG) performance status 2–3	12	32
Esophagus (*n*)	3	3
Colorectal (*n*)	15	25
Pancreas, gallbladder, biliary tract (*n*)	23	29
Breast (*n*)	11	11
Lung (*n*)	7	13
Brain (*n*)	1	1
Haematologic (*n*)	9	7
Kidney and urinary tract (*n*)	14	15
Others (*n*)	12	10
Metastatic disease (*n*)	60	88
Chemotherapy (*n*)	77	97
Radiotherapy (*n*)	17	22
Pulmonary embolism (*n*)	51	45
Deep vein thrombosis (*n*)	15	32
PICC-DVT (*n*)	13	19
VTE-A (*n*)	16	18

*PICC-DVT* peripherally inserted central catheter-deep venous thrombosis, *VTE-A* atypical venous thromboembolism

**Table 2:** Clinical characteristics of the four treatment subgroup

	Enoxaparin (*n* = 45)	Fondaparinux (*n* = 69)	Rivaroxaban (*n* = 27)	Edoxaban (*n* = 68)
Male (*n*)	21	41	16	34
Female (*n*)	24	28	12	33
Mean age (years)	67.8 ± 9.2	68.5 ± 10	68.2 ± 11	69.8 ± 10.3
Body weight (Kg)	77.5 ± 32.5	74.3 ± 30.1	79.7 ± 31.5	78.1 ± 33.4
Follow-up (months)	6 ± 6	6 ± 6	6 ± 6	6 ± 6
Eastern cooperative oncology group (ECOG) performance status 0–1	26	53	25	54
Eastern cooperative oncology group (ECOG) performance status 2–3	17	15	2	11
Esophagus (*n*)	1	2	3	0
Colorectal (*n*)	11	14	3	12
Pancreas, gallbladder, biliary tract (*n*)	8	21	7	16
Breast (*n*)	5	6	1	10
Lung (*n*)	3	10	3	4
Brain (*n*)	0	1	1	0
Haematologic (*n*)	4	3	2	7
Kidney and urinary tract (*n*)	8	7	3	11
Others (*n*)	5	5	5	7
Metastatic disease (*n*)	36	52	18	42
Chemotherapy (*n*)	38	59	21	56
Radiotherapy (*n*)	5	17	4	13
Pulmonary embolism (*n*)	12	20	5	10
Deep vein thrombosis (*n*)	18	27	16	35
PICC-DVT (*n*)	10	9	4	9
VTE-A (*n*)	5	13	2	14

*PICC-DVT* peripherally inserted central catheter-deep venous thrombosis, *VTE-A* atypical venous thromboembolism

## Outcomes

The primary outcome was a composite of recurrent venous thromboembolism or major bleeding. Recurrent venous thromboembolism was defined in our study as symptomatic new deep-vein thrombosis or pulmonary embolism, incidental new deep-vein thrombosis or pulmonary embolism involving segmental or more proximal pulmonary arteries, or fatal pulmonary embolism or unexplained death for which pulmonary embolism could not be ruled out as the cause. Incidental venous thromboembolism was defined as thromboembolism as detected using imaging tests performed for reasons other than clinical suspicion of venous thromboembolism. As for the criteria of the International Society on Thrombosis and Haemostasis (ISTH), major bleeding was defined as overt bleeding that was associated with a decrease in the haemoglobin level of 2 g per decilitre or more, led to a transfusion of 2 or more units of blood, occurred in a critical site, or contributed to the death of the patient (Schulman and Kearon 2005).

Secondary outcomes were mortality and clinically relevant non-major bleeding (CRNMB) defined as any sign or symptom of haemorrhage (e.g. more bleeding than would be expected for a clinical circumstance, including bleeding found by imaging alone) that does not fit the criteria for the ISTH definition of major bleeding but does meet at least 1 of the following criteria: requiring medical intervention by a healthcare professional or leading to hospitalization or increased level of care or prompting a face-to-face (i.e. not just a telephone or electronic communication) evaluation (Kaatz et al. 2015).

## Results

Table 3 shows the biochemical characteristics of the direct oral anticoagulants (DOAC) and heparin groups. A composite primary-outcome event occurred in 13 (11.4%) of 114 patients treated with heparins and in 10 (10.5%) of 95 treated with DOAC (Relative Risk 0.92; 95% CI 0.42–2.01, *p* = 0.84). We have recorded recurrent VTE in 7 patients (6.1%) in the heparins group and in 8 patients (8.4%) in the DOAC group (RR 1.37; 95% CI 0.51–3.64, *p* = 0.52). MB occurred in 6 patients (5.2%) in the heparins group and in 2 patients (2.1%) in the DOAC group (RR 0.40; 95% CI 0.08–1.93, p = 0.25). CRNMB were 13 (11.4%) in the heparins group and 15 (15.8%) in the DOAC group (RR 1.38; 95% CI 0.69–2.76, *p* = 0.36).

**Table 3:** Biochemical characteristics of the direct oral anticoagulants (DOAC) group and heparins one

	DOAC (*n* = 95)	HEPARINs (*n* = 114)	*p* value
Hemoglobin (g/dl)	11.8 ± 1.7	12 ± 1.7	0.91
Hematocrit (%)	36 ± 5	37 ± 4.8	0.67
Platelet count (× 10^3^)	223 ± 151	240 ± 114	0.89
Creatinine (mg/dl)	0.81 ± 0.3	0.82 ± 0.3	0.32
Prothrombin time (INR)	1.19 ± 0.2	1.11 ± 0.1	0.34
Activated partial thromboplastin time (INR)	1 ± 0.2	0.97 ± 0.2	0.38
Alanine aminotransferase (UI/l)	24 ± 15.4	22 ± 11.3	0.60
Aspartate aminotransferase (UI/l)	23.5 ± 22	23 ± 29.7	0.23

We observed 33 deaths (28.9%) in the heparins group and 17 (17.9%) in the DOAC group (Odds Ratio 0.53; 95% CI 0.27 to 1.04, *p* = 0.064). Below we will show the results obtained in the treatment subgroups (Table 4).

**Table 4:** Shows the endpoints in the treatment subgroups

	MB	re-VTE	Composite	CRNMB	Dead
Enoxaparin (*n* = 45)	2	0	2	4	16
Fondaparinux (*n* = 69)	4	7	11	9	17
Rivaroxaban (*n* = 27)	1	5	6	4	6
Edoxaban (*n* = 68)	1	3	4	11	11

*Re-VTE* recurrent venous thromboembolism, *MB* major bleeding, *CRNMB* clinical relevant non-major bleeding

**Graph 1:**
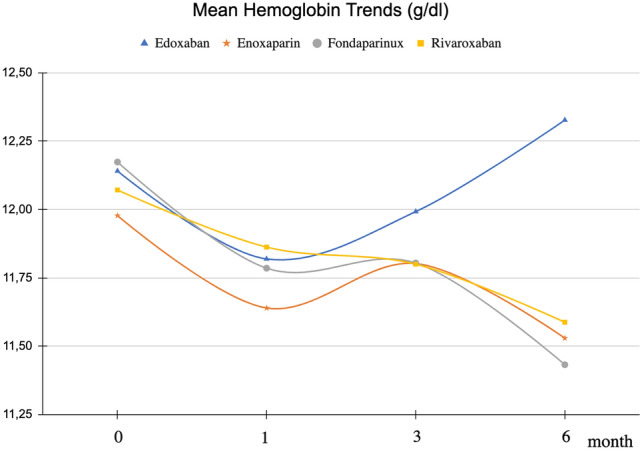
Hemoglobin trends in the treatment subgroups during the follow-up (6 months)

There were 2 deaths, 1 major bleeding and 0 recurrent VTE in patients taking edoxaban 30 mg. 2 deaths, 0 major bleeding and 1 recurrent VTE occurred in patients taking fondaparinux 5 mg. The haemoglobin baseline level was 12.17 ± 1.69 g/dl and 12.14 ± 1.79 g/dl (*p* = 0.91) in the fondaparinux and edoxaban treatment groups respectively. At 6 months from the start of therapy, haemoglobin values were 11.43 ± 1.9 in the fondaparinux group and 12.33 ± 1.79 in edoxaban patients (*p* = 0.06, if we consider haematocrit *p* = 0.05) (Graph 1). Haemoglobin values at 12 months were not shown due to insufficient data. Differences with other subgroups, in particular high bleeding risk vs other type of cancer, show no significant differences in mean haemoglobin trend over time.

6 MB and 17 CRNMB (of which 4 and 7 were on heparin therapy, respectively) occurred in the high risk bleeding neoplasms group (pancreas, biliary tract, gallbladder, oesophagus, colorectal and urinary tract). The haemoglobin value at the start of therapy was 11.94 ± 1.8 g/dl and 12.36 ± 1.61 g/dl (*p* = 0.09) in the high risk of bleeding cancer group and in the other cancer types group, respectively (Table 5).

**Table 5:** Shows haemoglobin changes (g/dL) in the high bleeding risk group and in the other cancers type group

	Baseline	1 month	3 month	6 month
High risk bleeding neoplasia	11.94 ± 1.8	11.63 ± 1.96	11.80 ± 1.86	11.55 ± 1.9
Other type of neoplasia	12.36 ± 1.61	11.98 ± 1.88	11.96 ± 1.88	12.11 ± 1.57
*p* value	0.09	0.22	0.60	0.13

None of the 20 patients tested for the plasma DOAC level resulted out of the therapeutic range at the nadir. We tested anti-factor Xa levels in 12 patients on edoxaban and 8 on rivaroxaban, respectively. All were on active chemotherapy treatment at the time of the blood draw which took place before the daily dose of the drug. The chemotherapeutics used are reported in Table 6.

**Table 6:** Shows the chemotherapeutics used in the patients in which we measured the anti-FXa activity

	Chemotherapy drugs
Edoxaban	Panitumumab
	Bevacizumab
	Nivolumab
	Ipilimumab
	Oxaliplatin
	Cisplatin
	5-fluorouracil
	Gemcitabine
	Paclixatel
	Pemetrexed
	Irinotecan Hydrochloride
	Octreotide
	Megestrol acetate
	Eribulin
Rivaroxaban	5-fluorouracil
	Oxaliplatin
	Cisplatin
	Gemcitabine
	Capecitabine
	Irinotecan hydrochloride
	PARP inhibitor
	Bevacizumab
	Cetuximab
	Ramucirumab

## Discussion

CAT is an important and frequent complication in the natural history of cancer, particularly during chemotherapy. The increased survival of these patients due to new therapeutic approaches has significantly increased the frequency of these episodes. Therefore it is fundamental to have pharmacological approaches able to prevent or treat effectively and safely these complications.

The results of our study confirm the equivalence of DOAC in terms of safety and efficacy compared to heparins even in a very heterogeneous sample of patients, types of cancer and thrombotic events. No significant DOAC-chemotherapy (strong P-glycoprotein and tyrosine kinase inhibitor, CYP3A4 competitor/inductor) interaction was noted, as suggested by the plasmatic dosage of drugs always in therapeutic range, confirming the possibility to use DOAC even during chemotherapy protocols. Adequate anticoagulant treatment for CAT lasts for at least 6 months and often exceeds a year. Also for this reason, compared with subcutaneous approaches, DOAC seem to be the preferred drugs in this clinical context with indisputable advantages in terms of therapeutic adherence and quality of life. The main limitation of our studio is its open-label protocol. The relatively high number of VTE recurrences in the rivaroxaban group could be explained by the need for food to ensure proper drug absorption (Stampfuss et al. 2013) and by the fact that eating habits can radically change during chemotherapy (Kenneth Fearon et al. 2011). This possibility should be investigated in future studies. Another important limitation is the greater number of patients with ECOG-PS 2–3 in the heparin group which justifies the higher mortality rate in this group. According to the latest guidelines on CAT management, recommendations for the use of DOACs in the medium to long term are still very low, particularly in gastrointestinal and urinary tumours and concomitant chemotherapy. Consequently, today many patients needing CAT treatments are excluded from the opportunity of accessing oral therapy with DOAC. The results of this study support the choice of DOAC over traditional treatment of CAT events using heparins. In this study, edoxaban has shown an excellent efficacy and safety profile even in patients taking a reduced dose, confirming its favourable profile. Another point to highlight is that most episodes of pulmonary embolism occur asymptomatically or following particular procedures such as placement of the PICC, suggesting the opportunity for prophylaxis in particular clinical situations or considering the primary prevention as a strategy in this setting. From this point of view, the efficacy of the reduced dose of edoxaban or the prophylactic dose of apixaban and rivaroxaban should be considered and evaluated in these particular situations. Moreover, more than the type of drug to be used, we believe that keystones in the management of CAT are the knowledge of the characteristics of anticoagulants available, the close collaboration of different specialists, the correct classification of bleeding and thrombotic risks in the single patient during the different phases of the disease.

## Conclusions

Edoxaban and rivaroxaban seem to be as effective and safe as heparins in the treatment of CAT even in patients with cancer at high risk of bleeding, in PICC-DVT and in VTE-A. No significant DOAC-chemotherapy (strong P-glycoprotein and tyrosine kinase inhibitor, CYP3A4 competitor/inductor) interaction was noted in our study. Larger clinical trials are needed to evaluate the effectiveness and safety of DOAC in CAT management.

## Data Availability

The datasets analysed during the current study are available from the corresponding author on reasonable request.
